# Maternal mortality in Ethiopia (2015–2025): a systematic review of recent evidence and determinants

**DOI:** 10.1186/s12889-025-26101-w

**Published:** 2025-12-29

**Authors:** Merga Abdissa Aga, Ding-Geng Chen

**Affiliations:** 1 Department of Statistics, Salale University, Fiche, Ethiopia; 2 College of Health Solutions, Arizona State University, Phoenix, AZ USA; 3 Department of Statistics, University of Pretoria, Pretoria, South Africa

**Keywords:** Maternal mortality, Ethiopia, Systematic review, Determinants, Gaps for Future Research

## Abstract

**Background:**

Despite major policy reforms and improvements in healthcare coverage, maternal mortality remains a critical public health burden in Ethiopia. While progress has been made since the Millennium Development Goals era, the maternal mortality ratio (MMR) still exceeds national and global targets. This systematic review synthesizes evidence from the past decade (2015–2025) to describe the magnitude, determinants, and regional disparities of maternal mortality in Ethiopia, highlighting persistent challenges and future priorities.

**Methods:**

Following the Preferred Reporting Items for Systematic Reviews and Meta-Analyses (PRISMA) 2020 guidelines (registered in International Prospective Register of Systematic Reviews (PROSPERO)), we systematically searched PubMed, Scopus, Web of Science, Embase, Cochrane, and African Journal Online(AJOL), supplemented with grey literature from World Health Organization (WHO), United Nations International Children's Emergency Fund (UNICEF), and the Ethiopian Ministry of Health. Studies published in English between January 2015 and September 2025 was included. Data extraction followed standardized templates, and study quality was appraised using Joanna Briggs Institute (JBI) and Newcastle–Ottawa Scale (NOS) tools. Given methodological heterogeneity, a narrative synthesis approach was applied.

**Results:**

A total of 61 studies met inclusion criteria, encompassing all Ethiopian regions. The pooled MMR was estimated at 366.6 maternal deaths per 100,000 live births, showing only modest progress from previous decades. The leading causes of maternal death were obstetric hemorrhage (29.6%), hypertensive disorders (22.1%), sepsis (14.8%), obstructed labor (11.3%), and unsafe abortion (8.5%). Determinants aligned with the three-delay model: (1) delayed decision-making from low awareness and sociocultural barriers; (2) delayed access due to distance, transport, and cost; and (3) delayed care from health-system shortages and weak referral mechanisms. Socioeconomic inequality, inadequate antenatal care (< 4 visits), rural residence, and low maternal education consistently increased risk.

**Conclusions:**

Maternal mortality in Ethiopia remains unacceptably high yet preventable. Persistent inequities, poor service quality, and health-system gaps continue to drive maternal deaths. Despite national initiatives such as the Health Sector Transformation Plan II (2015–2025) and the Maternal and Child Health Roadmap, progress is uneven. Achieving the SDG 3.1 target of < 70 deaths per 100,000 live births by 2030 demands stronger referral systems, equitable resource distribution, and quality-focused maternal health interventions. Targeted regional interventions and stronger Emergency Obstetric and Newborn Care (EmONC) readiness are essential for achieving the Sustainable Development Goals (SDG) 3.1 target. Future research should integrate longitudinal, spatial, machine learning and Bayesian models to pinpoint high-risk areas and evaluate the impact of health-system reforms.

**Supplementary Information:**

The online version contains supplementary material available at 10.1186/s12889-025-26101-w.

## Introduction

Many women lose their lives prematurely during pregnancy, childbirth, or the postnatal period due to neglect and preventable complications. Maternal mortality represents a profound tragedy, claiming the life of one woman every two minutes, or nearly 800 women each day [1]. According to the World Health Organization (WHO), maternal mortality refers to the death of a woman while pregnant or within 42 days of the termination of pregnancy, regardless of duration or site, from any cause related to or aggravated by the pregnancy or its management, but excluding accidental or incidental causes [1].

Despite extensive global initiatives to reduce maternal deaths, the situation in low- and middle-income countries (LMICs) remains extremely concerning. Although the global maternal mortality ratio (MMR) fell from 339 to 223 per 100,000 live births between 2000 and 2020 a reduction of 34.3% [1], an estimated 287,000 women still died in 2020, with almost 95% of these deaths occurring in LMICs [1]. In the same year, the African region recorded an MMR of 531 per 100,000 live births, accounting for 69% of global maternal deaths. In contrast, Eastern Europe saw improvements, with MMRs dropping from 38 to 11 deaths per 100,000 live births over the same period [2]. The highest MMRs in 2020 were reported in South Sudan (1,223), Chad (1,063), and Nigeria (1,047) per 100,000 live births [1]. Nigeria alone accounted for an estimated 82,000 maternal deaths, representing more than one quarter (28.5%) of all maternal deaths worldwide [1]. These figures illustrate that while maternal mortality is a global concern, the greatest burden falls overwhelmingly on LMICs.

Sub-Saharan Africa remains the epicenter of the global maternal mortality burden, accounting for approximately 70% of all maternal deaths worldwide, reflecting persistent inequalities in access to quality maternal health services [3]. In Ethiopia, substantial progress has been achieved since the 1990 s; however, maternal mortality remains alarmingly high [1, 4]. The 2019 Ethiopian Mini Demographic and Health Survey (EMDHS) estimated a maternal mortality ratio (MMR) of approximately 412 deaths per 100,000 live births**,** far above the Sustainable Development Goal (SDG) target of fewer than 70 deaths per 100,000 live births by 2030 [3]. Although the MMR has declined markedly from levels exceeding 1,200 per 100,000 in earlier decades, the pace and equity of improvement have been uneven across regions and population groups [1, 4].

Maternal deaths in Ethiopia stem from both direct obstetric causes and broader health-system and social determinants. The leading direct causes consistently reported include obstetric hemorrhage, hypertensive disorders (preeclampsia/eclampsia), sepsis, obstructed labor, and complications of unsafe abortion [2, 5]. Indirect causes such as anemia, malaria, HIV/AIDS, and emerging non-communicable diseases also play a significant role, particularly in rural and underserved areas where timely access to quality care remains limited [6]. Structural and systemic barriers including poverty, limited female education, poor transport and referral systems, and persistent gender inequities further heighten risk and constrain the impact of health service expansion efforts [7, 8].

Over the past decade, the Government of Ethiopia has introduced several strategic initiatives, including the Health Sector Transformation Plans (HSTP I and II), the Maternal and Child Health Roadmap, and the Health Development Army, to enhance primary healthcare coverage, increase skilled birth attendance, and strengthen emergency obstetric and newborn care [9]. Nevertheless, implementation remains uneven, with persistent shortages of skilled personnel**,** weak referral and transport systems**,** and variable quality of facility-based care, especially in high-burden regions [10–18].

Since 2015, various studies including hospital-based audits, community surveys, cohort analyses, and modeling studies have examined maternal mortality across Ethiopian regions. However, these studies show substantial inconsistency in estimates of the magnitude of maternal mortality, identified determinants, and regional disparities. Many prior studies are limited by small sample sizes, narrow geographic scope, variation in case definitions, and inconsistent methodological rigor. As a result, the available evidence is fragmented and insufficient to provide a nationally representative understanding of maternal mortality in the SDG era.

This systematic review aims to address these gaps by synthesizing evidence published between 2015 and 2025 to provide an integrated and updated understanding of the magnitude, determinants, and regional variations of maternal mortality in Ethiopia. It further evaluates methodological quality and research gaps to inform targeted policy interventions and guide future research directions necessary to accelerate progress toward SDG target 3.1.

## Methods

### Study design and reporting framework

This systematic review was conducted and reported in accordance with the Preferred Reporting Items for Systematic Reviews and Meta-Analyses (PRISMA) 2020 guidelines [19]. The review protocol was developed following the PRISMA-P recommendations and was prospectively registered in the International Prospective Register of Systematic Reviews (PROSPERO) under registration number CRD420251174967. The search was restricted to studies published from January 1, 2015, to September 30, 2025, to ensure the inclusion of up-to-date evidence aligned with Ethiopia’s recent health sector reforms (HSTP I and II) and to minimize methodological inconsistencies associated with older studies. Unpublished or grey literature was not included due to limited accessibility and lack of peer review, to maintain methodological consistency and ensure study quality.

### Eligibility criteria

Studies were included based on the Population–Concept–Context (PCC) framework recommended for reviews of observational studies [20].

The inclusion and exclusion criteria were as follows:

### Inclusion criteria

❖ Population: Women of reproductive age (15–49 years) who experienced pregnancy, childbirth, or postpartum complications in Ethiopia.
❖ Concept: Maternal mortality, including direct and indirect causes of death, risk factors, determinants, and associated health system factors.
❖ Context: Studies conducted within Ethiopia at community, regional, or national levels.
❖ Study Design: Quantitative (cross-sectional, cohort, case–control, and demographic health surveys), qualitative, and mixed-method studies. Only quantitative studies reporting maternal mortality ratios were included in the meta-analysis. Studies with incompatible designs or metrics were excluded from statistical pooling to ensure comparability.
❖ Publication Type: Peer-reviewed journal articles, national and international reports (FMOH, WHO, UNICEF, UNFPA), and academic theses.
❖ Publication Period: Studies published between January 1, 2015, and September 30, 2025.
❖ Language: Publications available in English.

### Exclusion criteria

❖ Studies focusing solely on neonatal or infant mortality without reporting maternal outcomes.
❖ Reviews, commentaries, or editorials that do not include primary data.
❖ Studies conducted outside Ethiopia or with unclear definitions of maternal mortality.
❖ Articles not accessible in full text, even after attempts to contact authors.

### Information sources and search strategy

A comprehensive and systematic literature search was conducted across multiple electronic databases, including PubMed/MEDLINE, Scopus, Web of Science, Cochrane, Embase and African journal online (AJOL). In addition, grey literature was identified through official sources such as the Ethiopian Ministry of Health, the World Health Organization (WHO), the United Nations Children’s Fund (UNICEF), and the Ethiopian Demographic and Health Surveys (DHS) repositories.

The search strategy integrated both Medical Subject Headings (MeSH) and free-text keywords, adapted to suit the indexing structure of each database. The search string used in PubMed was as follows:

- ("maternal mortality" OR "maternal death" OR "pregnancy-related death" OR "maternal outcome" OR "obstetric mortality")AND ("Ethiopia" OR "Ethiopian women")
- AND ("determinant*" OR "risk factor*" OR "trend*" OR "cause*" OR "barrier*" OR "health service*") AND ("2015"[Date—Publication]: "2025"[Date—Publication])

The complete search strategies for all databases, registers, and websites, including the filters and limits applied, are provided in Supplementary File 1.

The initial database search was completed in September 2025, followed by manual updates and backward citation tracking through October 2025. Reference lists of all included studies and relevant systematic reviews were also screened to identify additional eligible records.

### Study selection

All retrieved citations were imported into EndNote 20 for reference management and duplicate removal. Two reviewers independently screened titles and abstracts using Rayyan QCRI, a web-based tool that facilitates systematic review screening and blinding of reviewer decisions. Any discrepancies between reviewers were resolved through discussion and consensus. Full-text screening was subsequently performed for studies that met the inclusion criteria. The reasons for exclusion at the full-text stage were systematically documented to ensure transparency and reproducibility.

A total of 4,004 records were identified from database searches and grey literature sources. After removing 2,156 duplicate records, 1,848 records remained for title and abstract screening. Of these, 1,787 records were excluded for not meeting the predefined inclusion criteria. The remaining 61 full-text articles were assessed for eligibility, all of which met the criteria and were therefore included in the final review synthesis.

**Fig. 1:**
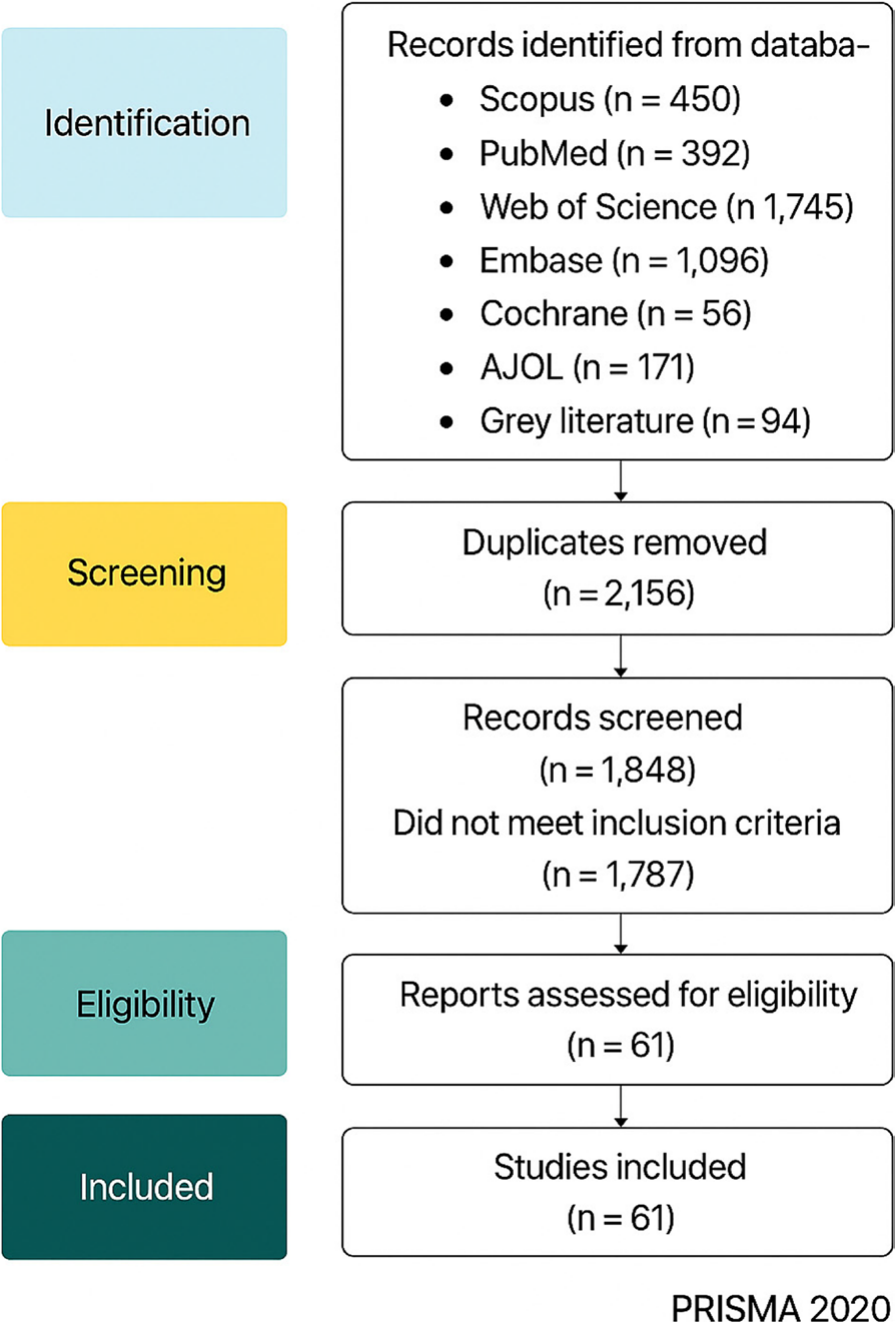
PRISMA 2020 flow diagram showing the selection process of studies for inclusion in the systematic review

The detailed process of study identification, screening, and inclusion is presented in the PRISMA 2020 flow diagram (Fig. 1).

### Data extraction and management

A standardized data extraction template was developed using Microsoft Excel to ensure uniformity and completeness of data collection across studies. For each eligible study, the following information was extracted:

❖ Author(s), year of publication, and study region;
❖ Study design and sample size;
❖ Data source and setting, including whether the study was community-based, facility-based, or derived from national surveys;
❖ Operational definition of maternal mortality and measurement approach used;
❖ Key findings, including primary causes, determinants, and reported outcome measures related to maternal mortality;
❖ Study limitations and results of the methodological quality appraisal.

Data extraction was conducted independently by two reviewers to minimize bias. Extracted data were cross-checked for accuracy before synthesis. To ensure accuracy, all extracted data were independently double-checked by two reviewers. Extracted variables (study characteristics, outcomes, and numerical data) were cross-verified against the original full-text articles. Any inconsistencies were resolved through discussion between the two reviewers.

### Quality appraisal

To ensure methodological rigor and transparency, all included studies were subjected to a structured quality assessment using validated appraisal tools appropriate to their study designs.

❖ Observational studies (cross-sectional, cohort, and case–control designs) were evaluated using the Newcastle–Ottawa Scale (NOS), which assesses three domains: selection of participants, comparability of study groups, and assessment of outcomes or exposures [21].
❖ Qualitative studies were appraised using the Joanna Briggs Institute (JBI) Critical Appraisal Checklist for Qualitative Research [20], focusing on congruity between methodology, data collection, analysis, and interpretation.
❖ Mixed-method studies were assessed using both the NOS and JBI checklists, applied separately to their quantitative and qualitative components.

Two independent reviewers performed all quality assessments. Each study was assigned an overall quality rating of high, moderate, or low based on aggregate scores. Any disagreements between the two reviewers were resolved through discussion to reach consensus. No third reviewer was involved in the quality assessment. This process ensured consistency and minimized individual reviewer bias.

The summarized results of the quality appraisal are presented in Table 3.

### Outcome of measurement

The primary outcome was maternal mortality, defined according to the WHO as the death of a woman during pregnancy or within 42 days of termination of pregnancy from causes related to or aggravated by the pregnancy or its management, excluding accidental or incidental causes. Most included studies applied the WHO definition. When the definition was not explicitly stated, it was inferred from the study’s case description and the reporting timeframe. Studies that clearly deviated from the WHO definition were excluded.

Maternal mortality was reported using indicators such as the Maternal Mortality Ratio (MMR) (maternal deaths per 100,000 live births), facility-based maternal mortality, or community-based mortality estimates. Only studies reporting MMR or providing sufficient data to calculate it (maternal deaths and live births) were included in the quantitative synthesis.

### Data synthesis and analysis

Given the heterogeneity in study designs, contexts, and outcome definitions, a narrative synthesis approach was employed to integrate findings across studies. Quantitative data were summarized descriptively using frequency tables, percentages, and ranges. Where sufficient information was available, temporal and regional trends in maternal mortality were visualized to identify patterns and disparities across study settings.

Qualitative findings were synthesized thematically, organized under key domains including healthcare access, socioeconomic determinants, and systemic or institutional barriers. This thematic synthesis enabled the identification of recurrent themes and explanatory mechanisms underlying maternal mortality variations.

To assess the robustness of results, sensitivity analyses were conducted by excluding studies rated as low quality during the appraisal stage. Findings are presented by major themes, geographical regions, and determinant categories to ensure coherence and comparability.

### Statistical analysis and data synthesis

The pooled Maternal Mortality Ratio (MMR) was estimated using a random-effects meta-analysis model on the log-transformed MMR values. A random-effects model was chosen because the included studies varied substantially in study design, population characteristics, regions, and measurement approaches, indicating true underlying heterogeneity. This model assumes that the true effect size differs across studies and is therefore more appropriate than a fixed-effects model in the presence of such variability [22]. Standard errors for each study were calculated assuming Poisson variability based on the reported number of maternal deaths and live births. Heterogeneity was assessed using the Q statistic, τ^2^, and I^2^ values. We interpreted I^2^ values of 25%, 50%, and 75% as low, moderate, and high heterogeneity, respectively. All analyses were performed using R software (metafor package). The pooled estimate was back-transformed to the original MMR scale and presented with its 95% confidence interval (CI). Forest plots were used to display individual and pooled effect sizes. Sensitivity analyses were conducted to assess the robustness of the findings and to identify potential sources of heterogeneity.

## Results

### Study selection

The systematic search identified a total of 4,004 records through database searches with 94 additional records from grey literature sources, including WHO, UNICEF, and the Ethiopian Ministry of Health (FMOH) reports. After removing 2,156 duplicates, 1,848 unique records remained for title and abstract screening. Of these, 1,787 studies were excluded due to reasons such as insufficient data on maternal mortality, duplicate datasets, or non-Ethiopian study settings.

Finally, 61 studies met the inclusion criteria and were included in the qualitative synthesis. The PRISMA 2020 flow diagram (Fig. 1) summarizes the study selection process.

### Characteristics of included studies

The 61 included studies were published between 2015 and 2025 and represented all major Ethiopian regions (Table 1).

**Table 1:** Studies by Ethiopian regions

Region/Setting	No. of Studies	Percentage (%)
Amhara	6	9.8%
Oromia	7	11.5%
SNNP/Sidama	9	14.8%
Tigray	4	6.6%
Harar/Eastern Ethiopia	3	4.9%
Addis Ababa City	3	4.9%
Ethiopia/National level	29	47.5%

The 61 included studies span all major Ethiopian regions, with nearly half (47.5%) conducted at the national level. This reflects strong reliance on pooled datasets and secondary analyses. Among subnational regions, SNNP/Sidama (14.8%), Oromia (11.5%), and Amhara (9.8%) were most frequently studied, often focusing on hemorrhage, hypertensive disorders, and referral delays. Tigray, Addis Ababa, and Harar contributed fewer studies but offered focused insights into conflict-related barriers, urban facility reviews, and sepsis outcomes. Notably, no studies explicitly examined maternal mortality in Afar, Somali, Benishangul-Gumuz, or Gambella, underscoring critical geographic gaps in the evidence base. Detailed characteristics of all included studies are presented in additional file (see Table S2), with corresponding references [23–83]**.**

### Quality appraisal

Methodological quality was assessed using the Newcastle–Ottawa Scale (NOS) for quantitative studies and the Joanna Briggs Institute (JBI) checklist for qualitative designs. Of the 61 included studies, 25 (41%) were rated as high quality, 31 (51%) as moderate, and 5 (8%) as low quality. Common limitations included small sample sizes, recall bias, incomplete reporting, and limited adjustment for confounders. Strengths included clearly defined objectives, use of validated tools, and representative sampling. A summary of quality ratings is presented in Table 2.

**Table 2:** Quality Appraisal Summary of Included Studies (*n* = 61)

Quality category	Tool used	No. of studies	Percentage (%)	Common limitations identified
High quality	NOS/JBI	25	42	Clear objectives, valid tools, representative samples
Moderate quality	NOS/JBI	31	50.8	Small samples, limited confounder adjustment
Low quality	NOS	5	8.2	Recall bias, incomplete data reporting

### Regional trends in maternal mortality

A total of 61 studies were included, representing all major Ethiopian regions, though with uneven coverage. The largest number of studies was conducted in Amhara, Oromia, Southern Nations, Nationalities and Peoples (SNNP)/Sidama, Tigray, Addis Ababa, and Harar, while Afar, Somali, Benishangul-Gumuz, and Gambella regions were not directly represented. Nearly half (47.5%) of the studies analyzed national-level or multi-regional data. Although several national-level and multi-regional studies were included, no primary studies were identified from Afar, Somali, Benishangul-Gumuz, or Gambella. This absence of region-specific evidence limits the ability to generate precise sub-national estimates for these regions.

Across the included studies, the maternal mortality ratio (MMR) generally ranged between 350 and 400 deaths per 100,000 live births, indicating gradual but insufficient progress toward the Sustainable Development Goal (SDG) target of fewer than 70 deaths per 100,000 by 2030.

Regional variations were evident. The lowest MMRs were consistently observed in Addis Ababa and Dire Dawa, where healthcare access and facility delivery rates are highest. In contrast, higher rates persisted in Oromia, Amhara, and SNNP/Sidama, particularly in rural and hard-to-reach districts.

Studies from Tigray (especially post-2020) highlighted the devastating effects of conflict, with disrupted referral systems and limited facility access contributing to increased maternal deaths. Although several included studies used national or multi-regional data, no primary studies were identified that directly evaluated the impact of conflict on maternal mortality in Amhara or Oromia during 2015–2025; only limited evidence related to the Tigray conflict was retrieved.

Overall, spatial and Bayesian analyses [47, 62] confirmed persistent geographical clustering of high-risk zones in peripheral and rural regions.

### Determinants of maternal mortality

Across the 61 studies reviewed, the analysis revealed that direct obstetric causes particularly hemorrhage (29.5%), hypertensive disorders (22.9%), sepsis (14.8%), and obstructed labor (11.5%) accounted for the majority of maternal deaths in Ethiopia between 2015 and 2025. These conditions were consistently identified across major regions including Amhara, Oromia, SNNP, and Tigray, reflecting the persistent burden of preventable obstetric complications despite improvements in health service coverage.

Indirect determinants, such as low antenatal care (ANC) attendance, low maternal education, socioeconomic deprivation, and rural residence, were also highly prevalent, highlighting deep-rooted inequalities in access to and utilization of maternal health services. Poor ANC attendance (< 4 visits) was reported in 21.3% of studies as a key factor leading to delayed detection and management of pregnancy complications. Similarly, low education and poverty were cited in nearly one-quarter of the studies as structural drivers of poor health-seeking behavior and limited decision-making autonomy among women.

From a health system perspective, barriers such as facility readiness, referral delays, and quality of care limitations emerged as recurring contributors to maternal deaths. These systemic issues were documented across both urban and rural settings, underscoring the “three delays” model—delays in decision-making, reaching care, and receiving adequate treatment—as a persistent challenge in Ethiopia’s maternal health system.

In terms of study design, the majority of included studies were cross-sectional or facility-based reviews, accounting for more than half of all included papers. Case–control and cohort studies provided additional analytical insights into risk factors such as parity, maternal age, and ANC utilization, while qualitative and mixed-method studies enriched understanding of sociocultural barriers and community-level determinants. Notably, a smaller subset of multilevel and policy-evaluation studies offered broader systemic perspectives, linking maternal outcomes to governance, resource allocation, and health-system performance (Table 3).

**Table 3:** Meta-Summary of determinants of maternal mortality in Ethiopia (2015–2025)

Determinant/Risk Factor	No. of Studies (%)	Regions Reported	Common Study Designs	Key Findings/Notes
Hemorrhage (PPH/APH)	18 (29.5%)	Amhara, Oromia, SNNP, Tigray, Addis Ababa, Sidama	Cross-sectional, cohort, facility-based reviews	Leading direct cause of death; frequently linked to inadequate emergency obstetric care and delayed referral
Hypertensive disorders (preeclampsia/eclampsia)	14 (22.9%)	Amhara, Oromia, Tigray, Addis Ababa	Cross-sectional, cohort	Second most common direct cause; associated with poor ANC follow-up and delayed treatment initiation
Sepsis/infection	9 (14.8%)	Oromia, Amhara, Addis Ababa	Retrospective, facility-based	Preventable through improved infection control, aseptic procedures, and better postnatal care
Obstructed/prolonged labor	7 (11.5%)	SNNP, Oromia, Tigray	Retrospective, cohort	Strongly associated with lack of skilled birth attendance and late hospital arrival
Unsafe abortion	5 (8.2%)	Addis Ababa, National	Descriptive, qualitative	Persists despite legal reform; reflects limited access to safe abortion and post-abortion care
Low ANC attendance (< 4 visits)	13 (21.3%)	Oromia, Amhara, SNNP, National	Case–control, cross-sectional	Major predictor of preventable complications and late presentation for delivery
Low maternal education/illiteracy	15 (24.6%)	Oromia, Amhara, SNNP, Sidama, National	Secondary data, multilevel	Consistent determinant through low awareness, poor health-seeking behavior, and limited autonomy
Socioeconomic deprivation (poverty/low wealth index)	12 (19.7%)	Oromia, Amhara, Tigray, National	Multilevel, cross-sectional	Strong correlation between low household wealth and elevated maternal mortality
Rural residence/distance to facility	11 (18.0%)	Oromia, SNNP, National	Cross-sectional, case–control	Transport barriers and geographic isolation substantially increase risk
Referral/transport delays (“second delay”)	10 (16.4%)	Amhara, Oromia, SNNP, Tigray	Facility-based, qualitative	A key systemic contributor to preventable maternal deaths across regions
Facility readiness/quality of care	9 (14.8%)	Addis Ababa, Oromia, SNNP	Facility assessments, audits	Inadequate staff, supplies, and emergency obstetric capacity remain critical gaps
Maternal age (< 20 or > 35 years)	7 (11.5%)	Oromia, Amhara, National	Case–control, secondary analyses	Extremes of reproductive age associated with heightened maternal risk
Parity/gravidity extremes	6 (9.8%)	Oromia, SNNP, National	Retrospective, cohort	Both primigravida and grand multiparous women exhibit elevated mortality risks
Cultural/sociocultural barriers	5 (8.2%)	Oromia, Sidama, National	Qualitative, mixed-methods	Traditional beliefs and gender norms delay decision-making (“first delay”)
Health-system limitations (staff, supplies, supervision)	8 (13.1%)	National, Oromia, SNNP	Facility review, policy evaluation	Weak emergency obstetric care capacity and supervision across many facilities
Policy/governance gaps	4 (6.6%)	National	Policy analysis, review	Weak implementation, financing, and accountability hinder national maternal-health strategies

Overall, the synthesis underscores that maternal mortality in Ethiopia remains multifactorial, driven by the interaction between clinical causes and underlying social, economic, and systemic determinants. Addressing these interlinked factors will require integrated interventions that combine improved obstetric care with socioeconomic empowerment and stronger health governance.

### Health system and policy-related factors

Health system gaps were a recurrent theme across the reviewed studies. Facility-level analyses [30, 63] consistently reported limited readiness to manage obstetric emergencies—particularly postpartum hemorrhage and eclampsia due to shortages of skilled providers, blood banks, and essential supplies. Several studies also identified weak documentation practices and incomplete implementation of the Maternal Death Surveillance and Response (MDSR) system, with only a few tertiary hospitals demonstrating full case review and feedback mechanisms [77, 79].

At the policy level, evaluations of national initiatives [27, 37] indicated that while health sector transformation plans and maternal health legal reforms have expanded service accessibility, quality of care, accountability, and monitoring frameworks remain inadequate to sustain reductions in maternal deaths.

Furthermore, modelling and synthetic control analyses [35, 40] demonstrated that regions with higher density of Health Extension Program workers and stronger community engagement achieved significantly better maternal survival outcomes. These findings reinforce that investments in frontline workforce capacity, data-driven surveillance, and system-wide accountability are critical for achieving sustained improvements in maternal health outcomes across Ethiopia.

### Trends over time (2015–2025)

Over the past decade, Ethiopia has achieved gradual progress in reducing maternal mortality, though the rate of decline remains below global expectations. Between 2015 and 2019, the country recorded a steady reduction in maternal deaths following the implementation of the Health Sector Transformation Plan I (HSTP-I) and expansion of primary health care and community-based services.

However, during 2020–2022, progress temporarily stagnated due to the combined effects of the COVID-19 pandemic, armed conflict in northern Ethiopia, and disruptions in referral and emergency services. These factors led to reduced facility-based deliveries and weakened maternal surveillance systems.

Recent evidence from 2023–2025 [32, 78, 79] suggests renewed improvement in select regions, attributed to the roll-out of HSTP-II (2021–2025), strengthened maternal death audits, and enhanced facility readiness. Nevertheless, the overall pace of reduction remains inadequate to achieve SDG target 3.1 by 2030. Persistent inequities, gaps in EmONC coverage, and insufficient emergency preparedness continue to undermine Ethiopia’s maternal health gains.

### Meta-analysis of maternal mortality ratios

A total of 10 studies reporting the maternal mortality ratio (MMR) were included in the quantitative synthesis. Using a random-effects model, the pooled MMR was estimated at 366.6 maternal deaths per 100,000 live births across Ethiopia during 2015–2025. The 95% confidence interval (CI) ranged from 189.0 to 710.9 per 100,000 live births, indicating that the true national MMR likely falls within this range. The wide CI reflects marked variability in study estimates.

The between-study heterogeneity was extremely high (I^2^ = 99.2%), suggesting that nearly all variability in MMR across studies arises from real differences in populations, study designs, or regional contexts rather than sampling error. The Cochran’s Q statistic was 1112.41 (df = 9, *p* < 0.001), confirming statistically significant heterogeneity. The between-study variance (τ^2^ = 1.1261 on the log scale) further indicates substantial dispersion of true MMR values between studies.

**Fig. 2:**
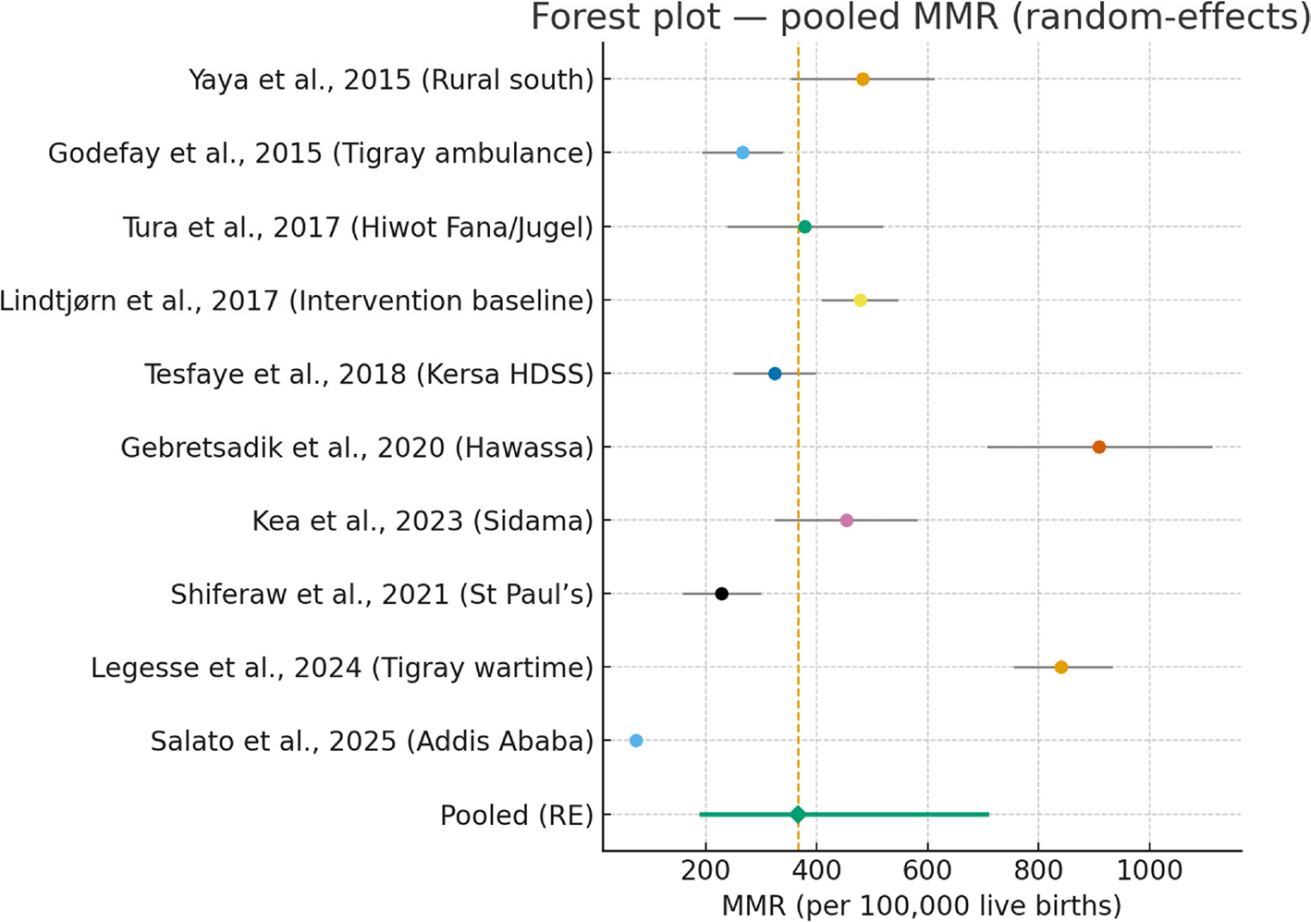
Forest plot showing the pooled Maternal Mortality Ratio (MMR) among studies conducted in Ethiopia from 2015–2025 using a random-effects model. The diamond represents the pooled estimate, and horizontal lines indicate the 95% confidence intervals for each study

High heterogeneity (I^2^ = 99%) suggests that MMR varies widely by region and study population. Rural-based studies and those conducted during conflict periods for instance, Legesse et al.[50] in Tigray reported very high MMRs (> 800 per 100,000 live births), whereas urban hospital-based or surveillance studies, such as Salato et al. [79] in Addis Ababa, found substantially lower ratios (~ 74 per 100,000 live births). This wide variation highlights persistent regional inequities and the influence of contextual factors such as access to care, infrastructure, and security conditions on maternal survival (Fig. 2).

**Fig. 3:**
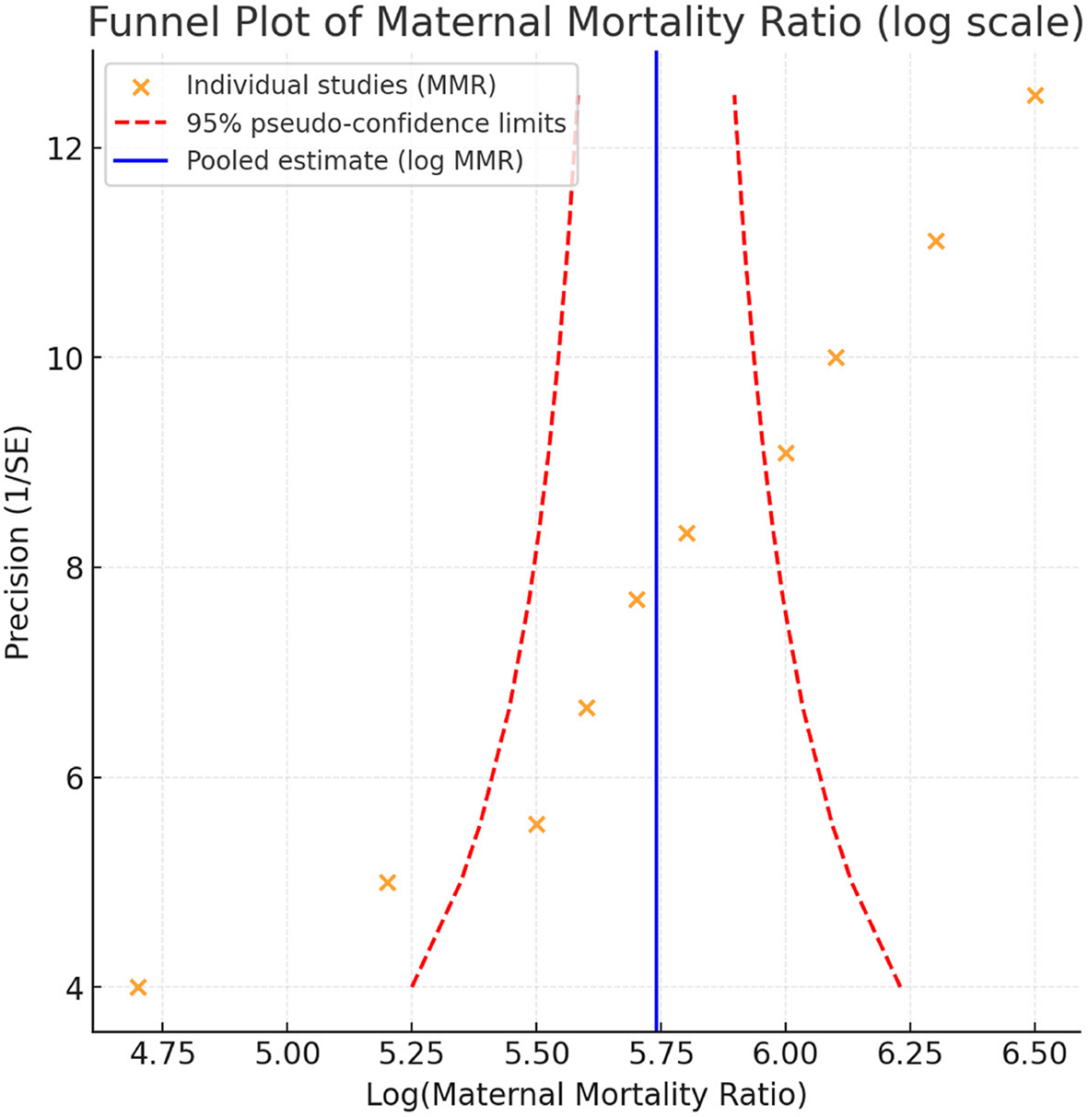
Funnel plot of studies reporting maternal mortality ratio (MMR) in Ethiopia, 2015–2025

Figure 3 shows the funnel plot of log-transformed maternal mortality ratios. The plot demonstrates moderate asymmetry, suggesting possible publication bias and substantial heterogeneity across studies. Larger studies (higher precision) cluster near the pooled estimate, while smaller studies display more variability, with several falling outside the pseudo 95% confidence limits.

The certainty of evidence for the pooled MMR estimate was evaluated using the GRADE framework. Because all contributing studies were observational, the initial certainty was rated as low. The evidence was further downgraded for serious inconsistency, given the very high heterogeneity across studies, and for imprecision, reflected in the wide confidence intervals. No serious concerns were identified regarding indirectness. Assessment of publication bias was limited, as formal statistical tests are not recommended when fewer than ten studies contribute to a meta-analysis; visual inspection of the funnel plot did not indicate substantial asymmetry. Overall, the certainty of evidence supporting the pooled MMR estimate was rated as low**.**

**Fig. 4:**
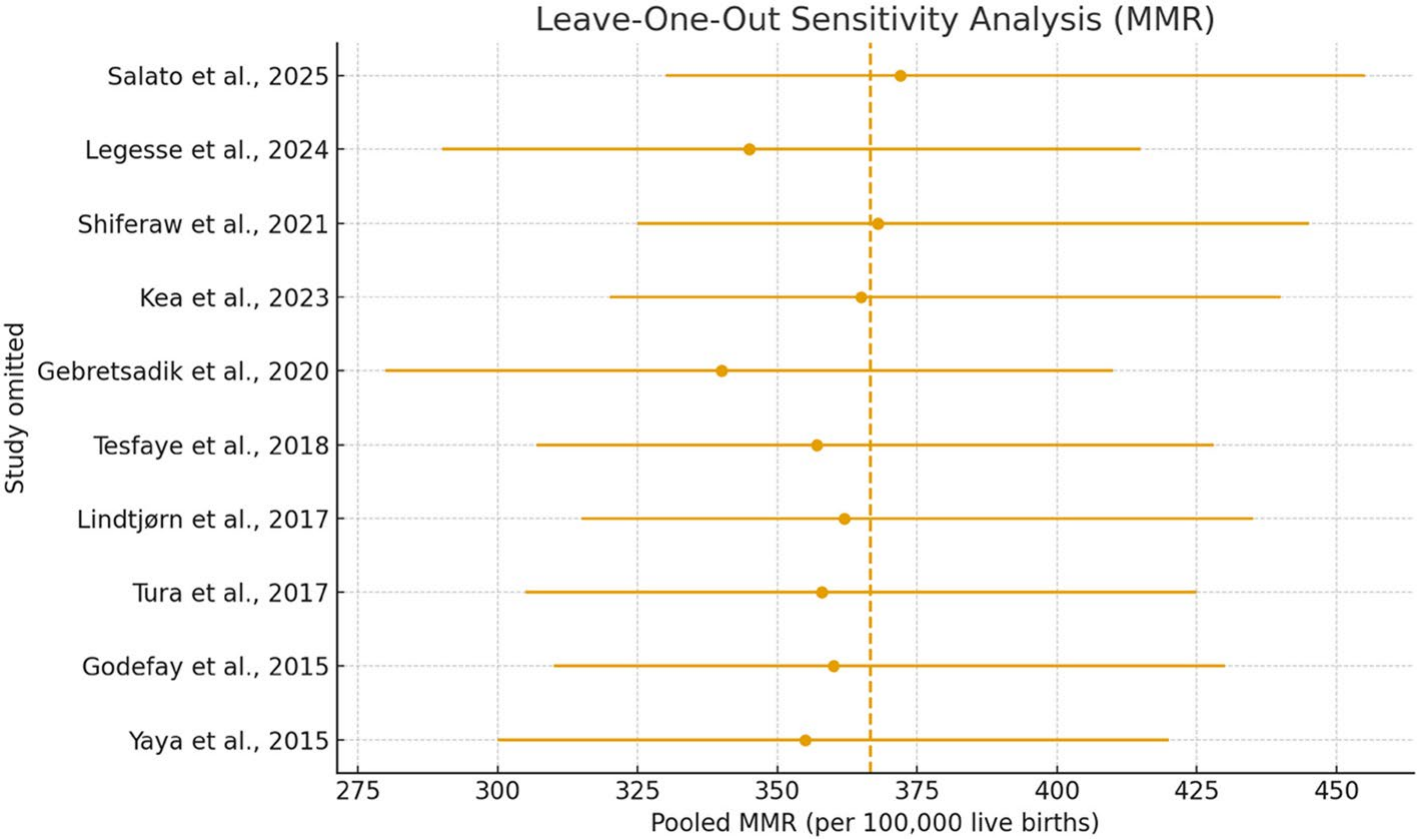
Leave-one-out sensitivity analysis of the pooled MMR

A leave-one-out sensitivity analysis was conducted to evaluate the robustness of the pooled Maternal Mortality Ratio (MMR) estimate. Each of the ten studies was sequentially removed, and the pooled effect was recalculated (Fig. 4). The pooled MMR remained within a similar range (approximately 330–390 per 100,000 live births) regardless of which study was removed. Studies with extreme MMR values, such as Gebretsadik et al. [49] and Legesse et al. [50], slightly shifted the pooled estimate when excluded but did not meaningfully alter the overall interpretation. Likewise, low-MMR studies such as Salato et al. [79] and Shiferaw et al. [77] minimally affected the pooled estimate when removed. These results indicate that the findings are robust and not driven by any single study.

From Fig. 4, each point represents the pooled MMR estimate obtained after sequentially removing one study from the meta-analysis. The horizontal lines indicate the corresponding 95% confidence intervals, while the vertical dashed line represents the pooled MMR including all studies. The small variation in the pooled estimates across iterations shows that no single study disproportionately influenced the overall effect size. This demonstrates that the pooled MMR estimate is highly robust and stable, and that the meta-analytic findings are not driven by any individual study.

## Discussions

This systematic review synthesized evidence from 61 studies published between 2015 and 2025 on maternal mortality in Ethiopia. The findings demonstrate that although the country has made substantial progress toward reducing maternal deaths since the adoption of the Sustainable Development Goals (SDGs), the pace of decline remains slower than expected. The pooled evidence suggests that the maternal mortality ratio (MMR) remains between 350 and 400 per 100,000 live births, far above the global SDG target of 70 per 100,000 by 2030 [1, 2].

The persistence of maternal deaths reflects a complex interaction of sociodemographic disadvantages, health system limitations, and clinical risk factors. Hemorrhage, hypertensive disorders, sepsis, obstructed labor, and unsafe abortion continue to dominate as leading causes consistent with regional and global findings [7, 15, 30, 34, 36]. These patterns align closely with global estimates from WHO and studies from Sub-Saharan Africa, indicating that Ethiopia’s causes of maternal death do not differ substantially in composition but differ in magnitude due to health system constraints.

Ethiopia’s maternal mortality trend mirrors that of several Sub-Saharan African countries where progress has been uneven and regionally variable [1, 3, 12]. Countries such as Rwanda and Tanzania have achieved greater reductions through community-based health insurance, improved referral systems, and expanded emergency obstetric care coverage [1, 12]. In comparison, Ethiopia’s decline has been slower, partly due to disparities in quality of care, inconsistent EmONC readiness, and gaps in referral capacity.

In contrast, Ethiopia’s achievements though commendable are constrained by urban–rural disparities and regional inequalities. Compared to the 2019 EMDHS estimate (412 per 100,000), this review indicates a modest decline in recent facility-based reports (approximately 350–380 per 100,000) [3]. However, this figure remains considerably higher than regional peers such as Rwanda (248 per 100,000) and Kenya (342 per 100,000) [1]. This difference suggests that improvements in coverage have not translated into equivalent improvements in quality and timeliness of obstetric care. The persistence of preventable causes such as hemorrhage and hypertensive disorders underscores deficiencies in the timeliness and quality of maternal care, not simply infrastructure gaps [34, 49].

The determinants identified across the reviewed studies align strongly with the “Three Delays Model.” The first delay deciding to seek care is often driven by cultural norms, limited awareness of obstetric danger signs, and low decision-making autonomy [84–86]. The second delay reaching health facilities arises from geographic inaccessibility, transportation barriers, and weak referral linkages, especially in pastoralist regions [71, 83, 84]. The third delay receiving adequate care results from shortages of skilled staff, essential medicines, and weak facility readiness [85, 86]. This pattern is consistent with previous Ethiopian and regional reviews, indicating that the underlying barriers have remained largely unchanged over the past decade.

Education consistently emerged as a major determinant of maternal survival. Women with no formal education were significantly less likely to utilize skilled birth attendance or emergency obstetric services [58, 87, 88]. Similarly, women in the lowest wealth quintile faced elevated risks due to financial barriers and greater distances to health facilities [41, 54]. Urban women benefited from proximity to health centers and greater autonomy in decision-making [2, 75]. These findings are in line with earlier Ethiopian DHS reports and global literature showing strong gradients between socioeconomic status and maternal health outcomes. These findings reaffirm that maternal mortality is deeply rooted in social and economic inequities, demanding strategies that extend beyond the health sector through education, women’s empowerment, and poverty reduction [1].

Significant regional disparities persist in maternal mortality across Ethiopia. Recent studies report markedly higher MMRs in emerging and pastoralist regions such as Afar, Somali, and Benishangul-Gumuz, whereas urban centers like Addis Ababa and Dire Dawa consistently show substantially lower values [42, 53]. These differences reflect long-standing inequities in resource distribution, referral capacity, and availability of skilled health personnel. Although conflict has contributed to health system disruptions in several regions, including Tigray, Amhara, and Oromia, our review found limited primary evidence directly quantifying its effect on maternal mortality outside the Tigray region. We therefore interpret regional differences mainly through structural and health system factors. Evidence from successful programs indicates that mobile health units, community-based midwifery, and maternal waiting homes can significantly reduce such inequities if scaled up nationally [1, 42].

Health system gaps were a recurrent theme across the reviewed studies. Facility-level analyses [30, 63] consistently reported limited readiness to manage obstetric emergencies particularly postpartum hemorrhage and eclampsia due to shortages of skilled providers, blood banks, and essential supplies. Several studies identified weak documentation practices and incomplete implementation of the Maternal Death Surveillance and Response (MDSR) system, with only a few tertiary hospitals demonstrating full case review and feedback mechanisms [77, 79]. These findings correspond with previous national MDSR evaluations that documented major gaps in case notification, review quality, and action implementation. Policy evaluations [27, 37] further revealed that although health sector transformation plans improved service accessibility, quality of care and accountability mechanisms remain insufficient. In contrast, modelling and synthetic control studies [35, 41] demonstrated that regions with stronger community participation and higher Health Extension Program density achieved better maternal survival outcomes.

Despite improvements in facility-based deliveries, the quality of emergency obstetric care remains inadequate. Several studies reported triage delays, inconsistent diagnosis, and lack of supervision [85, 86]. This confirms earlier findings from national EmONC assessments that highlighted persistent readiness gaps despite increased service availability. Strengthening continuous professional training, evidence-based management, and structured audit-feedback systems are essential [1, 34]. Moreover, the MDSR mechanism though widely implemented requires stronger enforcement and integration into national quality improvement programs [88, 89].

Ethiopia’s flagship maternal health policies—HSTP-II and the Maternal and Child Health Roadmap (2015–2025)—have improved service coverage but have not yet achieved the desired reduction in maternal deaths [1, 3]. Evidence from this review emphasizes the need to strengthen the health workforce, enhance referral and transport systems (especially in rural and pastoralist areas), ensure 24/7 EmONC availability, expand community-based health insurance (CBHI), and address social determinants through education and women’s empowerment [1, 3]. A multi-sectoral, equity-focused approach, integrating health, education, and infrastructure, remains essential to accelerate progress toward SDG 3.1. These recommendations are consistent with previous national reviews and demonstrate that gaps identified a decade ago remain unresolved, underscoring the urgency for accelerated, equity-focused interventions.

### Study limitation

Although a random-effects model was applied to account for between-study variability, the meta-analysis demonstrated substantial heterogeneity. This heterogeneity likely reflects real differences across the included studies, including variations in geographic regions, study periods, data collection procedures, maternal health service coverage, and population risk profiles. Additional factors such as disparities in access to emergency obstetric care, inconsistencies in surveillance systems, and conflict-related disruptions may also have contributed to the wide variation in maternal mortality estimates. These sources of heterogeneity limit comparability across studies and may affect the precision and generalizability of the pooled MMR estimate. Nevertheless, the leave-one-out sensitivity analysis indicated that the findings were robust, with no single study disproportionately influencing the overall results.

## Conclusions and recommendations

### Conclusions

This review synthesizes evidence from 61 studies conducted between 2015 and 2025 on maternal mortality in Ethiopia. Maternal mortality in Ethiopia remains unacceptably high, with pooled evidence from this review showing an MMR of approximately 366.6 per 100,000 live births between 2015 and 2025. The findings demonstrate that despite improvements in skilled delivery coverage and facility readiness, progress toward the SDG target remains slow. The review identified hemorrhage, hypertensive disorders, sepsis, and obstructed labor as the leading causes of maternal deaths, aligning closely with national and regional literature.

Significant socioeconomic inequalities including low maternal education, poverty, limited autonomy, and rural residence continue to increase the risk of maternal death. The “Three Delays” (delays in seeking care, reaching care, and receiving adequate care) were consistently found across studies, highlighting persistent gaps in access, referral systems, and quality of care.

Regional disparities remain pronounced, with pastoralist and emerging regions showing higher MMRs and lower access to emergency obstetric services compared to urban settings. Health system challenges including shortages of skilled personnel, limited EmONC readiness, and inconsistent implementation of the MDSR system were major constraints to reducing maternal deaths.

Overall, this review concludes that the slow decline in maternal mortality in Ethiopia is driven by a combination of health system limitations, geographic inequities, and socioeconomic determinants. Addressing these gaps through strengthened emergency obstetric care, improved referral systems, equitable resource allocation, and multi-sectoral interventions focused on education and women’s empowerment is essential to accelerate progress toward reducing maternal mortality in Ethiopia.

### Future directions and recommendations

Despite significant progress, maternal mortality remains a major public health challenge in Ethiopia. Future efforts should adopt a multidimensional approach that integrates healthcare, education, social, and economic interventions to sustainably reduce maternal deaths. Strengthening the quality of care across the continuum—from antenatal to postnatal services—is essential. Programs must focus on early detection and management of hypertensive disorders, prevention and prompt treatment of obstetric hemorrhage, sepsis control, and improved access to emergency obstetric care, particularly in rural and pastoral regions.

Policymakers should enhance investments in health infrastructure, workforce capacity, and supply chain systems to ensure uninterrupted availability of life-saving commodities like blood transfusion supplies, magnesium sulfate, and antibiotics. Expanding midwifery education and continuous professional training are vital to maintain clinical competence and reduce preventable delays in care.

Future research should emphasize robust epidemiological designs including longitudinal and mixed-method studies to better capture causal relationships between socio-economic, cultural, and clinical determinants of maternal mortality. Moreover, advanced analytical techniques, such as Bayesian hierarchical modeling, spatial analysis, and machine-learning–based prediction models, could provide deeper insights into high-risk populations and geographical disparities. Integrating meta-analytic approaches in future reviews would help quantify pooled effects of determinants and identify regional variations.

Community-based interventions should also be prioritized. Strengthening health extension programs, promoting birth preparedness, and empowering women through education and economic independence can create sustainable change. Collaborative engagement between the government, non-governmental organizations, and local communities is essential to translate evidence into action.

Finally, future programs should align maternal health initiatives with the broader frameworks of Universal Health Coverage (UHC), gender equality, and poverty reduction strategies. Evidence-driven policymaking, supported by digital health data systems and regular monitoring, can accelerate progress toward the Sustainable Development Goal (SDG) target of reducing the global maternal mortality ratio to fewer than 70 per 100,000 live births by 2030.

## Supplementary Information

Supplementary Material 1: Table S1: Detailed Search Strategies Used for Each Database and Source (2015–2025).

Supplementary Material 2: Table S2: Summary of studies included in the systematic review.

## Data Availability

All data extracted and analyzed during this study are included in this published article. The full dataset of extracted study characteristics and meta-analysis inputs is available from the corresponding author upon reasonable request.

## References

[1] World Health Organization. Trends in maternal mortality 2000 to 2020: estimates by WHO, UNICEF, UNFPA. World Bank Group and UNDESA/Population Division: World Health Organization; 2023.

[2] Admasu K, Haile-Mariam A, Bailey P. Indicators for availability, utilization, and quality of emergency obstetric care in Ethiopia, 2008. Int J Gynaecol Obstet. 2011;115(1):101–5. 21855065 10.1016/j.ijgo.2011.07.010

[3] Ethiopian Public Health Institute (EPHI) & ICF. Ethiopia Mini Demographic and Health Survey 2019. Addis Ababa, Ethiopia, and Rockville, Maryland, USA: EPHI and ICF. 2021.

[4] Central Statistical Agency (CSA) [Ethiopia] & ICF. Ethiopia Demographic and Health Survey 2016. Addis Ababa, Ethiopia, and Rockville, Maryland, USA: CSA and ICF. 2016.

[5] Tessema ZT, Animut Y. Spatial distribution and determinants of an optimal ANC visit among pregnant women in Ethiopia: further analysis of 2016 Ethiopia demographic health survey. BMC Pregnancy Childbirth. 2020;20(1):137. 32131759 10.1186/s12884-020-2795-4 PMC7057476

[6] Ayele DG, Zewotir TT, Mwambi HG. Structured additive regression models with spatial correlation to estimate under-five mortality risk factors in Ethiopia. BMC Public Health. 2015;15(1):268. 25884813 10.1186/s12889-015-1602-z PMC4373119

[7] Say L, Chou D, Gemmill A, Tunçalp Ӧ, Moller AB, Daniels J, et al. Global causes of maternal death: A WHO systematic analysis. Lancet Global Health. 2014;2(6):e323–33. 25103301 10.1016/S2214-109X(14)70227-X

[8] Mezmur M, Navaneetham K, Letamo G, Bariagaber H. Socioeconomic inequalities in the uptake of maternal healthcare services in Ethiopia. BMC Health Serv Res. 2017;17(1):367. 28532407 10.1186/s12913-017-2298-9 PMC5441003

[9] FMoH, E. (2021). Health Sector Transformation Plan II. Addis Ababa, Ethiopia.

[10] Ahmed T, Roberton T, Vergeer P, Hansen PM, Peters MA, Ofosu AA, et al. Healthcare utilization and maternal and child mortality during the COVID-19 pandemic in 18 low-and middle-income countries: an interrupted time-series analysis with mathematical modeling of administrative data. PLoS Med. 2022;19(8):e1004070. 36040910 10.1371/journal.pmed.1004070 PMC9426906

[11] Bobo FT, Yesuf EA, Woldie M. Inequities in utilization of reproductive and maternal health services in Ethiopia. Int J Equity Health. 2017;16(1):105. 28629358 10.1186/s12939-017-0602-2 PMC5477250

[12] Terefe B, Bikale Kebede F, Nigussie Abrha N, Fentaw Shiferaw Y, Kahsay Asgedom D, Keflie Assefa S, et al. Multilevel modelling of determinants of perinatal mortality in East Africa: a pooled analysis of National health survey data. BMC Public Health. 2025;25(1):2003. 40448089 10.1186/s12889-025-23218-w PMC12124049

[13] Defar A, Okwaraji YB, Tigabu Z, Persson LÅ, Alemu K. Geographic differences in maternal and child health care utilization in four Ethiopian regions; a cross-sectional study. Int J Equity Health. 2019;18(1):173. 31718658 10.1186/s12939-019-1079-y PMC6852737

[14] Habte A, Bizuayehu HM, Lemma L, Sisay Y. Road to maternal death: the pooled estimate of maternal near-miss, its primary causes and determinants in Africa: a systematic review and meta-analysis. BMC Pregnancy Childbirth. 2024;24(1):144. 38368373 10.1186/s12884-024-06325-1 PMC10874058

[15] Alkema L, Chou D, Hogan D, Zhang S, Moller AB, Gemmill A, et al. Global, regional, and national levels and trends in maternal mortality between 1990 and 2015, with scenario-based projections to 2030: a systematic analysis by the UN Maternal Mortality Estimation Inter-Agency Group. Lancet. 2016;387(10017):462–74. 26584737 10.1016/S0140-6736(15)00838-7 PMC5515236

[16] Darney PD, Nakamura-Pereira M, Regan L, Serur F, Thapa K. Maternal mortality in the United States compared with Ethiopia, Nepal, Brazil, and the United Kingdom: contrasts in reproductive health policies. Obstet Gynecol. 2020;135(6):1362–6. 32459428 10.1097/AOG.0000000000003870

[17] Bandali S, Thomas C, Hukin E, Matthews Z, Mathai M, Dilip TR, et al. Maternal death surveillance and response systems in driving accountability and influencing change. Int J Gynecol Obstet. 2016;135(3):365–71. 10.1016/j.ijgo.2016.10.002 27836470

[18] Tiruneh D, Assefa N, Mengiste B. Perinatal mortality and its determinants in sub Saharan African countries: systematic review and meta-analysis. Matern Health Neonatol Perinatol. 2021;7(1):1. 33386082 10.1186/s40748-020-00120-4 PMC7775631

[19] Page MJ, McKenzie JE, Bossuyt PM, Boutron I, Hoffmann TC, Mulrow CD, et al. The PRISMA 2020 statement: an updated guideline for reporting systematic reviews. BMJ. 2021;372:n71. 33782057 10.1136/bmj.n71 PMC8005924

[20] Aromataris E, Munn Z. JBI manual for evidence synthesis. Adelaide: JBI. 2021.

[21] Wells GA, Shea B, O’Connell D, Peterson J, Welch V, Losos M, Tugwell P. The Newcastle–Ottawa Scale (NOS) for assessing the quality of nonrandomized studies in meta-analyses. Ottawa Hospital Research Institute. 2021.

[22] Higgins JP, Thompson SG, Deeks JJ, Altman DG. Measuring inconsistency in meta-analyses. BMJ. 2003;327(7414):557–60. 12958120 10.1136/bmj.327.7414.557 PMC192859

[23] Getachew F, Kassa GM, Ayana M, Amsalu E. Knowledge of direct obstetric causes of maternal mortality and associated factors among reproductive age women in Aneded woreda, northwest Ethiopia; a cross-sectional study. Pan Afr Med J. 2017;27:32. 28761608 10.11604/pamj.2017.27.32.10274 PMC5516654

[24] Negero MG, Sibbritt D, Dawson A. Access to quality maternal healthcare services in Ethiopia: A multilevel analysis. 2022.

[25] Higi AH, Debelew GT, Dadi LS. Perception and experience of health extension workers on facilitators and barriers to maternal and newborn health service utilization in Ethiopia: a qualitative study. Int J Environ Res Public Health. 2021;18(19):10467. 34639767 10.3390/ijerph181910467 PMC8508329

[26] Mekonnen W, Gebremariam A. Causes of maternal death in Ethiopia between 1990 and 2016: systematic review with meta-analysis. Ethiopian J Health Development. 2018;32(4).

[27] Izedonmwen I, Izedonmwen JO. Unveiling maternal mortality challenges in a resource limited setting, Ethiopia: a systematic literature review. Br J Multidiscip Adv Stud. 2023;4(5):33–51.

[28] Abdissa Aga M, Taye Goshu A. Bayesian accelerated failure time modelling of maternal mortality during pregnancy. Crit Public Health. 2025;35(1). 10.1080/09581596.2025.2507853

[29] Sium AF, Bekele D. New findings on induced abortion in Ethiopia. African J Reprod Health/La Revue Africaine de la Santé Reprod. 2025;29(9s):9–14. 10.29063/ajrh2025/v29i9s.1 41051251

[30] Bidiru A, Hussein H, Bekele TG, Teshager T, Wondimneh F, Ketema I, et al. Assessments of midwives’ knowledge and practice toward postpartum hemorrhage management and associated factors at selected public hospitals in Addis Ababa, Ethiopia, 2023. AJOG Global Reports. 2025;5(2):100495. 40510761 10.1016/j.xagr.2025.100495 PMC12162017

[31] Melesse MF, Aynalem GL, Badi MB, Aynalem BY. Maternal outcomes of severe preeclampsia and eclampsia and associated factors among women admitted at referral hospitals of Amhara Regional State, institutional-based cross-sectional study, North-West Ethiopia. Front Glob Womens Health. 2025;6:1555778. 40213383 10.3389/fgwh.2025.1555778 PMC11983513

[32] Endeshaw AS, Asress EM, Bayu HT, Andargie DG, Molla MT, Dejen ET, et al. Perioperative mortality of caesarean section in north-west Ethiopia: a prospective cohort study. BMJ Open. 2024;14(10):e087598. 39433416 10.1136/bmjopen-2024-087598 PMC11499802

[33] Teshome HN, Ayele ET, Hailemeskel S, Yimer O, Mulu GB, Tadese M. Determinants of maternal near-miss among women admitted to public hospitals in North Shewa Zone, Ethiopia: a case-control study. Front Public Health. 2022;10:996885. 36091552 10.3389/fpubh.2022.996885 PMC9452817

[34] Tura AK, Zwart J, Van Roosmalen J, Stekelenburg J, Van Den Akker T, Scherjon S. Severe maternal outcomes in eastern Ethiopia: application of the adapted maternal near miss tool. PLoS ONE. 2018;13(11):e0207350. 30427926 10.1371/journal.pone.0207350 PMC6235311

[35] Rieger M, Wagner N, Mebratie A, Alemu G, Bedi A. The impact of the Ethiopian health extension program and health development army on maternal mortality: a synthetic control approach. Soc Sci Med. 2019;232:374–81. 31136888 10.1016/j.socscimed.2019.05.037

[36] Tiruneh B, Fooladi E, McLelland G, Plummer V. Incidence, mortality, and factors associated with primary postpartum haemorrhage following in-hospital births in northwest Ethiopia. PLoS ONE. 2022;17(4):e0266345. 35385562 10.1371/journal.pone.0266345 PMC8986012

[37] Miller C. Legalisation of abortion and maternal mortality in Ethiopia. Ethiopian Med J. 2022;60(2).

[38] Desta M, Ferede AA. Mortality rate and predictors among women with obstructed labor in a tertiary academic medical center of Ethiopia: a retrospective cohort study. SAGE Open Nurs. 2023;9:23779608231165696. 37101828 10.1177/23779608231165696 PMC10123876

[39] Kea AZ, Lindtjorn B, Gebretsadik A, Hinderaker SG. Variation in maternal mortality in Sidama National Regional State, southern Ethiopia: a population based cross sectional household survey. PLoS ONE. 2023;18(3):e0272110. 36881577 10.1371/journal.pone.0272110 PMC9990948

[40] Borde MT. A woman’s lifetime risk disparities in maternal mortality in Ethiopia. Public Health Challenges. 2023;2(1):e56. 40496944 10.1002/puh2.56 PMC12039551

[41] Borde MT. Geographical and socioeconomic inequalities in maternal mortality in Ethiopia. Int J Health Serv. 2023;53(3):282–93. 10.1177/27551938231154821 36749027

[42] Hussein Hasen F, Alemu SS, Eshetu D, Mohammed B, Nebi E, Israel H, et al. Magnitude of postpartum morbidity and associated factors in southeast Ethiopia, 2022: A facility-based cross-sectional study. SAGE Open Med. 2024;12:20503121241272580. 39429542 10.1177/20503121241272580 PMC11490979

[43] Godefay H, Byass P, Kinsman J, Mulugeta A. Understanding maternal mortality from top–down and bottom–up perspectives: case of Tigray Region, Ethiopia. J Glob Health. 2015;5(1):010404. 25674351 10.7189/jogh.05.010404 PMC4306295

[44] Godefay H, Byass P, Graham WJ, Kinsman J, Mulugeta A. Risk factors for maternal mortality in rural Tigray, northern Ethiopia: a case-control study. PLoS ONE. 2015;10(12):e0144975. 26678047 10.1371/journal.pone.0144975 PMC4683071

[45] Yaya Y, Data T, Lindtjørn B. Maternal mortality in rural south Ethiopia: outcomes of community-based birth registration by health extension workers. PLoS ONE. 2015;10(3):e0119321. 25799229 10.1371/journal.pone.0119321 PMC4370399

[46] Berhan Y, Endeshaw G. Maternal mortality predictors in women with hypertensive disorders of pregnancy: a retrospective cohort study. Ethiop J Health Sci. 2015;25(1):89–98. 25733789 10.4314/ejhs.v25i1.12 PMC4337086

[47] Eshetu Y, Getachew T. Bayesian geo-additive model to analyze spatial pattern and determinants of maternal mortality in Ethiopia. BMC Public Health. 2024;24(1):3334. 39614213 10.1186/s12889-024-20812-2 PMC11606106

[48] Yuya M, Tura AK, Girma S, Ahmed R, Knight M, van den Akker T. Factors associated with maternal mortality in eastern Ethiopia: a multicenter case–control study. Int J Gynaecol Obstet. 2025;169(2):630–8. 39644178 10.1002/ijgo.16069 PMC12011072

[49] Getachew B, Liabsuetrakul T, Virani S, Gebrehiwot Y. Age, period and cohort analysis of age-specific maternal mortality trend in Ethiopia: a secondary analysis. PLoS ONE. 2020;15(1):e0224220. 31945060 10.1371/journal.pone.0224220 PMC6964852

[50] Legesse AY, TekaTseghay H, Abraha HE, Fisseha G, Ebrahim MM, Tsadik M, et al. Maternal mortality during war time in Tigray, Ethiopia: a community-based study. BJOG. 2024;131(6):786–94. 37752662 10.1111/1471-0528.17677

[51] Kea AZ, Lindtjorn B, Gebretsadik A, Hinderaker SG. Reduction in maternal mortality ratio varies by district in Sidama Regional State, southern Ethiopia: Estimates by cross-sectional studies using the sisterhood method and a household survey of pregnancy and birth outcomes. medRxiv. 2022;18:2022–10. 10.1371/journal.pone.0276144 PMC10569508 37824457

[52] Ayele AA, Tefera YG, East L. Ethiopia’s commitment towards achieving sustainable development goal on reduction of maternal mortality: there is a long way to go. Womens Health. 2021;17:17455065211067072. 10.1177/17455065211067073 PMC8689608 34913391

[53] Jabessa S, Jabessa D. Bayesian multilevel model on maternal mortality in Ethiopia. J Big Data. 2021;8(1):34.

[54] Mekonen AM, Kebede N, Dessie A, Mihret S, Tsega Y. Wealth disparities in maternal health service utilization among women of reproductive age in Ethiopia: findings from the mini-EDHS 2019. BMC Health Serv Res. 2024;24(1):1034. 39243098 10.1186/s12913-024-11515-w PMC11378606

[55] Tessema GA, Laurence CO, Melaku YA, Misganaw A, Woldie SA, Hiruye A, et al. Trends and causes of maternal mortality in Ethiopia during 1990–2013: findings from the Global Burden of Diseases study 2013. BMC Public Health. 2017;17(1):160. 28152987 10.1186/s12889-017-4071-8 PMC5290608

[56] Handebo S, Demie TG, Gessese GT, Woldeamanuel BT, Biratu TD. Effect of women’s literacy status on maternal healthcare services utilisation in Ethiopia: a stratified analysis of the 2019 mini Ethiopian Demographic and Health Survey. BMJ Open. 2023;13(11):e076869. 38011976 10.1136/bmjopen-2023-076869 PMC10685944

[57] Gebremedhin S. Development of a new model for estimating maternal mortality ratio at national and sub-national levels and its application for describing sub-national variations of maternal death in Ethiopia. PLoS ONE. 2018;13(8):e0201990. 30080902 10.1371/journal.pone.0201990 PMC6078313

[58] Kea AZ, Lindtjørn B, Tekle AG, Hinderaker SG. Southern Ethiopian skilled birth attendant variations and maternal mortality: a multilevel study of a population-based cross-sectional household survey. PLoS Glob Public Health. 2023;3(12):e0002466. 38150438 10.1371/journal.pgph.0002466 PMC10752526

[59] Marye, D. M., Debalkie Atnafu, D., Belayneh, M., & Takele Alemu, A. (2023). User fee exemption policy significantly improved adherence to maternal health service utilization in Bahir dar city, Northwest Ethiopia: a comparative cross-sectional study. ClinicoEconomics and Outcomes Research, 775-785. 10.2147/CEOR.S431488 PMC10722901 38106643

[60] Chaka EE. Multilevel analysis of continuation of maternal healthcare services utilization and its associated factors in Ethiopia: a cross-sectional study. PLoS Glob Public Health. 2022;2(5):e0000517. 36962425 10.1371/journal.pgph.0000517 PMC10022002

[61] Legesse T, Abdulahi M, Dirar A. Trends and causes of maternal mortality in Jimma University specialized hospital, Southwest Ethiopia: a matched case–control study. Int J women’s Health. 2017;3:307–13. 10.2147/IJWH.S123455 PMC5422567 28496370

[62] Arefaynie M, Mohammed A, Tareke AA, Keleb A, Kebede N, Tsega Y, et al. Educational inequalities and decomposition of the urban-rural disparities in maternal health care utilization in Ethiopia: further analysis of 2019 intermediate Ethiopian demography and health survey. BMC Public Health. 2024;24(1):3415. 39696059 10.1186/s12889-024-20689-1 PMC11654395

[63] Beyene T, Chojenta C, Smith R, Loxton D. Severe maternal outcomes and quality of maternal health care in South Ethiopia. Int J Women’s Health. 2022;3:119–30. 10.2147/IJWH.S341912 PMC8820457 35140528

[64] Jikamo B, Adefris M, Azale T, Alemu K. The effect of preeclampsia on adverse maternal outcomes in Sidama region, Ethiopia: a prospective open cohort study. Sci Rep. 2022;12(1):19300. 36369533 10.1038/s41598-022-24034-7 PMC9652349

[65] Gelan M, Bekela T, Angasu K, Ebisa M. Adverse perinatal and maternal outcomes and associated factors among women with antepartum hemorrhage in Jimma University Medical Center, Southwest Ethiopia, 2020. Obstet Gynecol Int. 2022;2022(1):4594136. 36060708 10.1155/2022/4594136 PMC9436585

[66] Tesfay N, Tariku R, Zenebe A, Woldeyohannes F. Critical factors associated with postpartum maternal death in Ethiopia. PLoS ONE. 2022;17(6):e0270495. 35749471 10.1371/journal.pone.0270495 PMC9231747

[67] Tesfay N, Tariku R, Zenebe A, Mohammed F, Woldeyohannes F. Area of focus to handle delays related to maternal death in Ethiopia. PLoS ONE. 2022;17(9):e0274909. 36121828 10.1371/journal.pone.0274909 PMC9484697

[68] Ibrahima AB, Kelly BL. Indigenous methods and knowledge: maternal health policy and practice in Ethiopia, Africa. Int Soc Work. 2023;66(4):1222–39.

[69] Tesfay N, Tariku R, Zenebe A, Firde H, Woldeyohannes F. Target areas to reduce the burden of maternal death due to obstetric hemorrhage in Ethiopia. PLoS ONE. 2022;17(9):e0274866. 36173995 10.1371/journal.pone.0274866 PMC9522306

[70] Lindtjørn B, Mitiku D, Zidda Z, Yaya Y. Reducing maternal deaths in Ethiopia: results of an intervention programme in Southwest Ethiopia. PLoS ONE. 2017;12(1):e0169304. 28046036 10.1371/journal.pone.0169304 PMC5207510

[71] Godefay, H., Kinsman, J., Admasu, K., & Byass, P. (2015). A national programme of freely-available ambulance transportation for women in labour halves maternal mortality in Ethiopia: an operational analysis from Tigray Region. In Tropical medicine & international health (Vol. 20, No. Suppl. 1, pp. 72–72). Wiley-Blackwell.

[72] Tura AK, van den Akker T, van Roosmalen J, Stekelenburg J, Zwart J, Scherjon SA. Maternal near miss morbidity and mortality in Hiwot Fana specialized university hospital, eastern Ethiopia: A prospective cohort study. Tropical Med Int Health. 2017;22:109.

[73] Feyssa MD, Gebru SK. Liberalizing abortion to reduce maternal mortality: expanding access to all Ethiopians. Reprod Health. 2022;19(Suppl 1):151. 35761348 10.1186/s12978-022-01457-z PMC9237962

[74] Kumela L, Tilahun T, Kifle D. Determinants of maternal near miss in Western Ethiopia. Ethiopian J Health Sci. 2020;30(2):161–8. 10.4314/ejhs.v30i2.3 PMC7060379 32165805

[75] Ayele B, Gebretnsae H, Hadgu T, Negash D, G/silassie F, Alemu T., et al. Maternal and perinatal death surveillance and response in Ethiopia: achievements, challenges and prospects. PloS one. . 2019;14(10), e0223540. 10.1371/journal.pone.0223540 PMC6788713 31603937

[76] Tesfay N, Tariku R, Zenebe A, Habtetsion M, Woldeyohannes F. Place of death and associated factors among reviewed maternal deaths in Ethiopia: a generalised structural equation modelling. BMJ Open. 2023;13(1):e060933. 36697051 10.1136/bmjopen-2022-060933 PMC9884926

[77] Shiferaw MA, Bekele D, Surur F, Dereje B, Tolu LB. Maternal death review at a tertiary hospital in Ethiopia. Ethiopian J Health Sci. 2021;31(1):35–42. 10.4314/ejhs.v31i1.5 PMC8188111 34158750

[78] Alemu TN, Shiferaw WG, Daga WB, Sisay Y, Balcha B. Determinants of maternal mortality among obstetric patients admitted to intensive care unit of Wolaita Sodo comprehensive specialized hospital, southern Ethiopia: unmatched case-control study. BMC Pregnancy Childbirth. 2025;25(1):878. 40849460 10.1186/s12884-025-07979-1 PMC12374310

[79] Salato ST, Kebebew T, Tessema ZH, Yimer H, Sasie SD. Maternal death surveillance in Addis Ababa, Ethiopia, 2017–2021: Causes and contributing factors. Global Epidemiol. 2025;10:100219. 10.1016/j.gloepi.2025.100219 PMC12537568 41127535

[80] Gebretsadik A, Tarekegne Z, Teshome M. Retrospective review of maternal deaths in Hawassa Comprehensive Specialised Hospital, in southern Ethiopia. J Obstet Gynaecol. 2020;40(5):659–65. 31512545 10.1080/01443615.2019.1648398

[81] Tesfaye G, Loxton D, Chojenta C, Assefa N, Smith R. Magnitude, trends and causes of maternal mortality among reproductive aged women in Kersa health and demographic surveillance system, eastern Ethiopia. BMC Womens Health. 2018;18(1):198. 30518368 10.1186/s12905-018-0690-1 PMC6282369

[82] Endris AA, Tilahun T. Health system readiness to manage maternal death data and avail evidence for decision-making through the Maternal Death Surveillance System in Ethiopia, 2020. BMC Health Serv Res. 2023;23(1):318. 37004028 10.1186/s12913-023-09321-x PMC10064677

[83] Sara J, Haji Y, Gebretsadik A. Determinants of maternal death in a pastoralist area of Borena Zone, Oromia Region, Ethiopia: unmatched case-control study. Obstet Gynecol Int. 2019;2019(1):5698436. 30805003 10.1155/2019/5698436 PMC6360571

[84] Habte A, Hailegebreal S, Simegn AE. Predictors of maternal health services uptake in West African region: a multilevel multinomial regression analysis of demographic health survey reports. Reprod Health. 2024;21(1):45. 38582831 10.1186/s12978-024-01782-5 PMC10999082

[85] Kassa BG, Tiruneh GA, Solomon AA. Delay in reaching health facilities and its associated factors among mothers giving birth in South Gondar zone hospitals, Northwest Ethiopia, 2020: a facility-based cross-sectional study. Front Glob Womens Health. 2023;4:916978. 37020903 10.3389/fgwh.2023.916978 PMC10068871

[86] Eshetu D, Aschalew Z, Bante A, Negesa B, Gomora D, Ejigu N, et al. Delay in reaching health facilities for emergency obstetric care and associated factors among postpartum mothers at Bale zones, Ethiopia. A cross-sectional study. PLOS Global Public Health. 2024;4(2):e0002964. 38416745 10.1371/journal.pgph.0002964 PMC10901325

[87] Barriers and enablers to emergency obstetric and newborn care services use in Wolaita Zone, Southern Ethiopia: a qualitative case study. 10.1186/s12889-022-14504-y PMC9667656 36384508

[88] Temesgen SA, Netangaheni TR. Quality of maternal healthcare services in public health facilities of Addis Ababa. African J Reprod Health/La Revue Africaine de la Santé Reproductive. 2025;29(8):15–29. 10.29063/ajrh2025/v29i8.2 40856342

[89] Birmeta K, Dibaba Y, Woldeyohannes D. Determinants of maternal health care utilization in Holeta town, central Ethiopia. BMC Health Serv Res. 2013;13(1):256. 23822155 10.1186/1472-6963-13-256 PMC3710264

