# Supplementary material for: Maternal mortality in Ethiopia (2015–2025): a systematic review of recent evidence and determinants
Source: BMC Public Health. 2025 Dec 29;26:539. doi: 10.1186/s12889-025-26101-w (PMC12888282; doi:10.1186/s12889-025-26101-w)
Supplement: Supplementary file 1 — Supplementary Material 1: Table S1: Detailed Search Strategies Used for Each Database and Source (2015–2025). [file 12889_2025_26101_MOESM1_ESM.docx]

**Table S1**. Detailed search strategies used for each database and source for the study

“Maternal Mortality in Ethiopia (2015–2025): A Systematic Review of Recent Evidence and Determinants”

| **Database / Source** | **Search Strategy (Exact Terms, Boolean Operators, Filters)** | **Limits / Filters Applied** | **Records Retrieved (n)** |
| --- | --- | --- | --- |
| PubMed | (“maternal mortality”[MeSH] OR “maternal deaths” OR “pregnancy-related mortality” OR “pregnancy-related death*” OR “obstetric mortality” OR “maternal fatal*”) AND (Ethiopia[MeSH] OR Ethiopia) | English; Humans; 2015–2025 | 392 |
| Embase | (‘maternal mortality’/exp OR ‘maternal death’ OR ‘pregnancy related death’ OR ‘obstetric death’ OR ‘maternal fatality’) AND (ethiopia:ab,ti OR 'ethiopia'/exp) | English; Humans; 2015–2025 | 1,096 |
| Cochrane Library | (“maternal mortality” OR “maternal death*” OR “pregnancy-related death*” OR “obstetric mortality”) AND (Ethiopia) | English;2015–2025 | 56 |
| Web of Science | TS = (“maternal mortality” OR “maternal death*” OR “pregnancy-related mortality” OR “obstetric death*”) AND TS = (Ethiopia) | English; 2015–2025 | 1,745 |
| Scopus | TITLE-ABS-KEY (“maternal mortality” OR “maternal death*” OR “pregnancy-related death*” OR “obstetric mortality”) AND TITLE-ABS-KEY (Ethiopia) | English; 2015–2025 | 450 |
| African Journals Online (AJOL) | “maternal mortality” AND Ethiopia; “maternal death*” AND Ethiopia; “pregnancy-related death*” AND Ethiopia | English; 2015–2025 | 171 |
| Grey Literature Sources (WHO, UNICEF, Ethiopian Ministry of Health) | Manual search using keywords: “maternal mortality Ethiopia”, “maternal death Ethiopia”, “pregnancy-related mortality Ethiopia”, “maternal health Ethiopia” | English; 2015–2025 | 94 |
| Total |  |  | **4,004** |
