# Supplementary material for: Maternal mortality in Ethiopia (2015–2025): a systematic review of recent evidence and determinants
Source: BMC Public Health. 2025 Dec 29;26:539. doi: 10.1186/s12889-025-26101-w (PMC12888282; doi:10.1186/s12889-025-26101-w)
Supplement: Supplementary file 2 — Supplementary Material 2: Table S2: Summary of studies included in the systematic review. [file 12889_2025_26101_MOESM2_ESM.docx]

Table S2: Summary of studies included in the systematic review

| **Author(s), Year** | **Region** | **Study Design** | **Key Determinants / Risk Factors** | **Main Findings** | **Quality Rating** |
| --- | --- | --- | --- | --- | --- |
| Getachew F. et al.[22] | Amhara | Cross-sectional | Knowledge/awareness, education, socioeconomic status, ANC attendance | Low knowledge of direct obstetric causes linked with lower service use and higher risk behaviours | Moderate |
| Negero MG, Sibbritt D, Dawson A.[23] | National / multi-region Ethiopia | Multilevel analysis or secondary data | Health facility access, socioeconomic status, facility quality, education | Access to quality maternal healthcare varies by cluster; individual and facility factors significant | Moderate |
| Higi AH et al.[24] | Ethiopia | Qualitative | Health extension worker capacity, community barriers, transport, cultural norms | HEWs report facilitators (community trust) and barriers (workload, resource gaps) to maternal/newborn service use | Low |
| Mekonnen W., Gebremariam A. [25] | Ethiopia | Systematic review & meta-analysis | Causes over time: hemorrhage, hypertensive disorders, sepsis, obstructed labour, abortion | Pooled causes and trends 1990–2016; confirm leading causes and reductions over time | High |
| Izedonmwen I., Izedonmwen JO.[26] | Ethiopia | Systematic literature review | Limited antenatal care services, rural residence, young maternal age, and a lack of education were linked to increased maternal death rates. The ongoing problem of maternal mortality in Ethiopia is highlighted by this systematic review. Even though there have been gains, particularly in metropolitan areas, | Limited antenatal care services, rural residence, young maternal age, and a lack of education were linked to increased maternal death rates. The ongoing problem of maternal mortality in Ethiopia is highlighted by this systematic review. Even though there have been gains, particularly in metropolitan areas, | Moderate |
| Abdissa Aga M., Taye Goshu A.[27] | Oromia | Retrospective | maternal age, fewer than four antenatal visits, later gestational age at admission, previous abortion and preeclampsia (current or historical) | maternal age, fewer than four antenatal visits, later gestational age at admission, previous abortion and preeclampsia (current or historical) | High |
| Sium AF., Bekele D.[28] | Ethiopia | Descriptive / review | Induced abortion incidence, access, safety | New findings on induced abortion patterns and implications for maternal mortality | Moderate |
| Bidiru A. et al.[29] | Addis Ababa city | Cross-sectional / facility assessment | Midwives’ knowledge/practice, training, availability of PPH supplies | Gaps in midwives’ PPH management knowledge/practice; associated factors include training and experience | Moderate |
| Melesse MF. et al.[30] | Amhara | cross-sectional | Severe preeclampsia/eclampsia, delayed referral, lack of ICU | Identifies predictors of poor maternal outcomes in severe preeclampsia/eclampsia patients | Moderate |
| Endeshaw AS. et al. [31] | Amhara | Prospective cohort | Surgical delays, anesthesia, emergency indications, comorbidities | Perioperative mortality after caesarean section quantified; system factors implicated | Moderate |
| Teshome HN. et al.[32] | Amhara | Case-control | Severe preeclampsia, postpartum hemorrhage, mothers education level, being referred from other health facilities, cesarean section, lack of ANC, medical disorder during pregnancy, Age | Severe preeclampsia, postpartum hemorrhage, mothers education level, being referred from other health facilities, cesarean section, lack of ANC, medical disorder during pregnancy, Age | Moderate |
| Tura AK. et al.[33] | Harar | Cross-sectional | Severe hemorrhage, hypertensive disorders, sepsis, referral delays | Reports rates of severe maternal outcomes and applied adapted MNM tool | Moderate |
| Rieger M. et al.[34] | Ethiopia | Quasi-experimental / synthetic control | Health extension program, Health Development Army (HDA) presence | HEP and HDA linked to reductions in maternal mortality where implemented | High |
| Tiruneh B. et al., [35] | Amhara | Cohort / incidence study | PPH risk factors: uterine atony, prolonged labour, retained placenta | Incidence and mortality from primary PPH after in-hospital births; identifies associated clinical predictors | Moderate |
| Miller C.[36] | Ethiopia | Policy / commentary | Abortion law/legalisation, access to safe services | Discusses link between abortion law changes and maternal mortality trends | Low |
| Desta M., Ferede AA. [37] | SNNP | Retrospective cohort | Obstructed labour, prolonged labour, fetal distress, delays, Homorrhage | Mortality rate and predictors among women with obstructed labour quantified, homorrhage | Moderate |
| Kea AZ. et al. [38] | SNNP | Population-based cross-sectional household survey | Geographic variation, access to skilled birth attendants, socioeconomic factors, Hemorrhage, educacion | Reports regional variation in maternal mortality within Sidama, Hemorrhage, educacion | High |
| Borde MT.[39] | Ethiopia | Secondary analysis / modelling | Lifetime risk disparities: region, SES, rural vs urban | Demonstrates disparities in lifetime maternal mortality risk across groups | High |
| Borde MT.[40] | Ethiopia | Cross-sectional / secondary analysis | Geographic location, education, income, health service access | Highlights geographic and socioeconomic inequalities in MMR | High |
| Hussein Hasen F. et al.[41] | Oromia | Cross-sectional | Postpartum morbidity predictors: obstetric complications, care delays, rular residence, unskilled birth | Magnitude of postpartum morbidity and associated factors reported, residence, unskilled birth | Moderate |
| Godefay H. et al.[42] | Tigray | Mixed methods / case studies | Socioeconomic, health system, cultural factors | Combines top-down and bottom-up perspectives to understand MMR in Tigray | Moderate |
| Godefay H. et al.[43] | Tigray | Case-control | Obstetric hemorrhage, hypertensive disorders, delayed care | Risk factors for maternal mortality in rural Tigray identified | Moderate |
| Yaya Y., Data T., Lindtjørn B.[44] | SNNP | Community-based birth registration analysis | Health extension worker coverage, birth practices, facility delivery | Community birth registration outcomes and maternal mortality patterns | High |
| Berhan Y., Endeshaw G.[45] | SNNP | Retrospective cohort | Hypertensive disorders severity, management gaps, referral, ANC, parity | Predictors of maternal mortality among women with HDP identified, ANC, parity | Moderate |
| Eshetu Y., Getachew T. [46] | Ethiopia | Cross sectional | Spatial clustering, facility density, rurality, SES, the place of delivery, the number of prenatal care visits, marital status, wealth index, mother's age and the number of birth orders | Maps spatial pattern of maternal mortality and determinants across Ethiopia, the place of delivery, the number of prenatal care visits, marital status, wealth index, mother's age and the number of birth orders | High |
| Yuya M. et al. [47] | Harar and Oromia | Multicenter case-control | Obstetric hemorrhage, hypertensive disorders, sepsis, delays | Identifies factors associated with maternal mortality in eastern Ethiopia | High |
| Getachew B. et al. [48] | Ethiopia | Secondary analysis | Age, period, cohort effects on maternal mortality | Provides APC decomposition of maternal mortality trends in Ethiopia | High |
| Legesse AY. et al. [49] | Tigray region | Community-based study | War-related access barriers, facility destruction, displacement, Haemorrhage, regnancy‐induced hypertension | Shows markedly increased MMR during wartime in Tigray, Haemorrhage, regnancy‐induced hypertension | High |
| Kea AZ. et al. [50] | Sidama | Cross-sectional | District variation, skilled birth attendance, facility access | Reduction in MMR varies by district; district-level estimates reported | High |
| Ayele AA., Tefera YG., East L. [51] | Ethiopia | Review | SDG commitment gaps, health system limitations | Argues Ethiopia still has a long way to achieve SDG 3.1 | Low–Moderate |
| Jabessa S., Jabessa D. [52] | Ethiopia | Cross sectional | Individual and cluster-level SES, education, facility variables, age of mother, marital status, number of living children, wealth index and Education | Multilevel determinants of maternal mortality modelled, age of mother, marital status, number of living children, wealth index and Education | High |
| Mekonen AM. et al. [53] | Ethiopia | Secondary data analysis | Wealth disparities, urban/rural, education, service use | Wealth-related disparities in maternal health service utilization identified | High |
| Tessema GA. et al.,[54] | Ethiopia | Secondary analysis | Cause trends over time: haemorrhage, HDP, sepsis | Trends and causes of maternal mortality (1990–2013) reported | High |
| Handebo S. et al. [55] | Ethiopia | Secondary analysis | Women’s literacy, education, health service utilization | Literacy positively affects maternal healthcare utilization | High |
| Gebremedhin S. [56] | Ethiopia | Cross sectional | Sub-national variation drivers: facility density, SES | New estimation model and demonstration of sub-national MMR variation | High |
| Kea AZ. et al. [57] | Sidama | Population-based cross-sectional household survey | Skilled birth attendant availability, district-level factors | Variation in skilled birth attendance and links to maternal mortality | High |
| Marye DM. et al. [58] | Amhara | Comparative cross-sectional | User fee exemption policy, service adherence, affordability | Fee exemption improved maternal service adherence in Bahir Dar | Moderate |
| Chaka EE.[59] | Ethiopia | Cross-sectional | Continuation of maternal services: education, distance, SES | Multilevel determinants of continued service utilization analysed | High |
| Legesse T. et al.[60] | Oromia | Matched case-control | Clinical causes, delays, referral system | Trends and causes of MMR in Jimma hospital examined | Moderate |
| Arefaynie M. et al.[61] | Ethiopia | Secondary analysis | Education inequalities, urban/rural disparities | Educational inequalities drive urban-rural disparities in maternal service use | High |
| Beyene T. et al.[62] | SNNP | Facility-based study (severe outcomes) | Quality of care, facility readiness, obstetric complications | Severe maternal outcomes linked to quality gaps in maternal healthcare | Moderate |
| Jikamo B. et al.[63] | Sidama region | Prospective cohort | Preeclampsia severity, ANC, referral timeliness | Effect of preeclampsia on adverse maternal outcomes reported | Moderate |
| Gelan M. et al.[64] | Oromia | Cross-sectional / facility-based | Antepartum hemorrhage, placenta praevia, hypertension | Adverse perinatal & maternal outcomes associated with APH detailed | Moderate |
| Tesfay N. et al.[65] | Ethiopia | Cross-sectional / case series | Postpartum critical factors: PPH, sepsis, delays | Critical factors associated with postpartum maternal death identified | Moderate |
| Tesfay N. et al.[66] | Ethiopia | Cross-sectional / analytical | First/second/third-delay factors: awareness, transport, facility care | Identifies areas to target for reducing delays related to maternal death | Moderate |
| Ibrahima AB., Kelly BL.[67] | Ethiopia | Review / policy analysis | Indigenous knowledge, cultural practices, policy alignment | Discusses integration of indigenous methods into maternal health policy | Low |
| Tesfay N. et al.[68] | Ethiopia | Cross-sectional / analytical | Obstetric hemorrhage drivers: ANC, facility readiness, PPH management | Target areas to reduce burden from obstetric hemorrhage identified | Moderate |
| Lindtjørn B. et al.[69] | SNNP | Intervention evaluation (before-after) | Community interventions, HEW program, facility strengthening | Intervention programme reduced maternal deaths in SW Ethiopia | High |
| Godefay H. et al.[70] | Tigray | Operational analysis / program evaluation | Free ambulance transport for women in labour | Ambulance program associated with halving maternal mortality in study area | High |
| Tura AK. et al.[71] | Harar | Prospective cohort | Severe complications, referral delays, facility factors | MNM morbidity and mortality rates and predictors presented | Moderate |
| Feyssa MD., Gebru SK.[72] | Ethiopia | Policy commentary / review | Legal access to abortion, safe services | Argues liberalised abortion can reduce maternal mortality | Low |
| Kumela L.et al. [73] | Oromia | Case-control | Severe complications, delays, ANC attendance | Determinants of maternal near miss in western Ethiopia identified | Moderate |
| Ayele B. et al.[74] | Ethiopia | Review | Surveillance system implementation, notification, response | Achievements and challenges of MPDSR in Ethiopia summarized | Moderate |
| Tesfay N. et al.[75] | Ethiopia | Cross sectional | Place of death determinants: facility vs home, delays, socio demographics | SEM identifies factors associated with place of maternal death | High |
| Shiferaw MA. et al. [76] | Addis Ababa city | review (facility-based) | Clinical causes, hypertensive disorders of pregnancy, postpartum hemorrhage, sepsis, quality of care, referral delays | Facility maternal death review findings and recommendations | Moderate |
| Alemu TN. et al.[77] | SNNP | Unmatched case-control | ICU admission predictors, severe obstetric complications | Determinants of maternal mortality among obstetric ICU patients | Moderate |
| Salato ST. et al. [78] | Addis Ababa city | Maternal death surveillance analysis | Contributing factors: PPH, sepsis, delays, referral failures | Causes and contributing factors of maternal deaths in Addis Ababa (2017–2021) | High |
| Gebretsadik A. et al. [79] | SNNP | Retrospective review | Clinical causes, referral delays, facility readiness | Retrospective maternal death review and cause distribution | Moderate |
| Tesfaye G. et al. [80] | Ethiopia | HDSS population-based analysis | Trends over time, causes: hemorrhage, hypertension, sepsis | Magnitude, trends and causes of MMR in Kersa HDSS reported | High |
| Endris AA., Tilahun T. [81] | Ethiopia | Health systems readiness assessment | Data systems, MDSR capacity, decision-making | Health system readiness to manage maternal death data assessed | Moderate |
| Sara J., Haji Y., Gebretsadik A.[82] | Oromia | Unmatched case-control | Distance, traditional birth practices, ANC, education | Determinants of maternal death in pastoralist area identified | Moderate |
